# High indoor temperatures increase reporting of acute symptoms: finding mitigating solutions for the climate-vulnerable of Bangladesh

**DOI:** 10.1186/s12889-025-21597-8

**Published:** 2025-06-06

**Authors:** Alba McGirr, Srizan Chowdhury, Md Fozla Rabbi, Md Mehedi Hasan, Md Sharoardy Sagar, Nabilah Ibnat, Syed Manzoor Ahmed Hanifi

**Affiliations:** 1 Department of Disease Control, Faculty of Infectious and Tropical Diseases, London School of Hygiene and Tropical Medicine, London, UK; 2 Health System and Population Studies Division, International Centre for Diarrhoeal Disease Research, Bangladesh (Icddr, B), Dhaka, Bangladesh; 3 Department of Bioengineering, George Mason University, Fairfax, VA USA

**Keywords:** Extreme temperatures, Heat index, Climate change, Acute symptoms, Mitigating measures, Vulnerable populations, Women, Rural

## Abstract

**Background:**

Bangladesh is already prone to extreme weather events like heatwaves, leaving millions vulnerable. High ambient temperatures are associated with increased morbidity and mortality by infectious diseases, but the effect of these high temperatures indoors remains to be studied.

**Objective:**

This study investigated the effect of high indoor temperatures on the feelings of illness and heat coping mechanisms in vulnerable populations without heat mitigation.

**Methods:**

A cross-sectional survey was conducted in 490 houses in rural villages in the coastal area of Chakaria, Bangladesh chosen through stratified cluster sampling. It assessed the feelings of illness and their adaptative behaviour to high temperatures. There were 49 temperature and humidity monitors placed indoors to obtain accurate measurements of these parameters in different areas and with different house materials. This information was used to determine the effect of high indoor temperatures on the symptoms that vulnerable populations reported.

**Results:**

People living in hotter houses reported overall more symptoms, notably, diarrhoea, local site infections and sore throat. Temperatures were higher in houses made of bamboo compared to cement and having shade significantly decreased indoor temperature. Most women in the study reported performing adequate heat coping mechanisms. However, these did not show a protective effect against illness.

**Conclusion:**

This paper showed that high indoor temperatures could be associated with an increase in symptoms. Housing characteristics (material and environment) decreased indoor temperature. Having shading and a house made from cement was protective to reporting symptoms. Further studies into the compliance of coping behaviours are needed to assess their potential protective effect.

## Introduction

Between 2000 and 2019, Bangladesh was the 7th most affected country by climate change [1]. The whole country is under high alert for heatwaves, cyclones and floods [2]. Experts point at raised temperatures and heat waves as one of the biggest health risks caused by climate change [3]. The effect of outdoor temperature with disease prevalence is well known. One study in Dhaka, Bangladesh, showed that rotavirus, the causative agent for 40% of child diarrhoea hospitalizations, increased its number of cases by 40% for every degree above the threshold of 29 °C [4]. Additionally, a variety of food-borne bacteria like Salmonella show increased expression of virulence genes under high temperatures [5].

Despite the relationship between many infectious diseases and ambient temperatures, few studies have been conducted to understand the specific effect of indoor temperatures. This question is of high relevance as worldwide people spend 70–80% of their time indoors [6]. Additionally, being indoors is known to be a coping mechanism to extreme heat as a study in rural Bangladesh that showed 90% of respondents did [7]. Unlike outdoor environments which are comparable for all, indoor temperatures are highly affected by housing characteristics which in turn is dependent on wealth. Considering this, it is crucial to understand how indoor temperatures could affect health, especially in those vulnerable people who cannot afford indoor heat mitigation in the form of fans or air conditioning.

This is especially a challenge for women. In Bangladesh and many other countries, women have a role in society which still largely remains as caregivers, leading them to spend long hours at home exposed to indoor temperatures [8]. Having the correct heat coping behaviours, like drinking more water or increasing ventilation, can increase the feeling of comfort and reduce heat burden [3]. Since not everyone has the same access to heat-mitigating housing characteristics and knowledge of heat-coping mechanisms, even where ambient temperatures are the same, people are affected differently by their indoor temperature. In this context, it is crucial to understand the extent to which it is possible to mitigate the negative effects of high indoor temperatures.

The present study aims to investigate the effects of high indoor temperature on prevalence of acute symptoms on rural Bangladeshi populations. It explores possible mitigating factors like heat coping mechanisms and housing characteristics for highly vulnerable populations, with a focus on women.

## Methods and materials

### Study site and design

International Center for Diarrheal Diseases Research, Bangladesh (icddr,b) runs a Health and Demographic Surveillance System (HDSS) in Chakaria. The HDSS covers a population of 90,000 individuals living in 16,000 households in 49 villages. Chakaria has a lower population density than the national average, 782 compared to 939 individuals/km2. Near the sea, livelihoods are gained by salt and shrimp production, whereas the hillier areas are greener and more prone to farming [9].

**Fig. 1:**
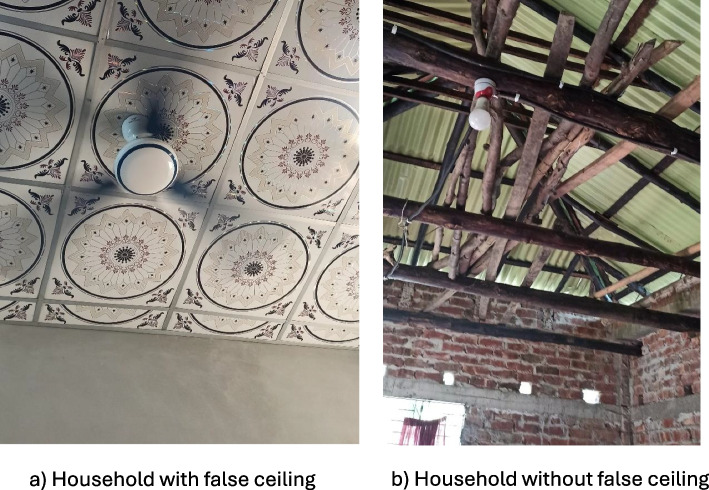
Image showing houses with and without a false ceiling

This cross-sectional study surveyed 490 households chosen through stratified cluster sampling. There were 49 clusters of 10 households chosen in each of the 49 villages from the HDSS. All 10 households in the village had to have the same housing characteristics to ensure indoor temperature data from the central house with the data logger was applicable to all. According to the HDSS, in this region the most common wall/roof combination was bamboo/tin and cement/tin and these were found throughout all the HDSS region and throughout all socio-economic strata. The villages were categorized depending on whether they had more bamboo-tin or cement-tin houses. In order to get an accurate representation of the population’s house characteristics, the number of villages with each material were chosen following the known proportions of household material from the HDSS data. In HDSS data about 65% of houses had bamboo walls and 35% had cement walls. Therefore, in this study 31 villages out of the 49 which were selected to include only houses with bamboo walls within that village and 18 villages which would select houses with cement walls. All households with the chosen household characteristics for that village were mapped and the household in the centre was used to store the logger, the 9 closest houses around it were then selected for the questionnaire. Information on whether they had a false ceiling which is a secondary ceiling below the primary ceiling used as an additional barrier from the roof, was also collected (Fig. 1).

In each house which was selected, health and background information of all the household members was collected. To be eligible, the participant had to live in a household with the right roof/wall materials and consent to be part of the study.

No acute symptom reporting could be found for Chakaria to be used for the sample size calculation. Therefore, mortality data was used which predicted 22% of deaths occurred by communicable diseases [10]. Using a level of confidence of 95% and margin of error of 5%, the sample size obtained was 263. To allow for more precision between symptom types, the sample size was increased to 490 households.

### Environmental information

#### Devices and installation

**Fig. 2:**
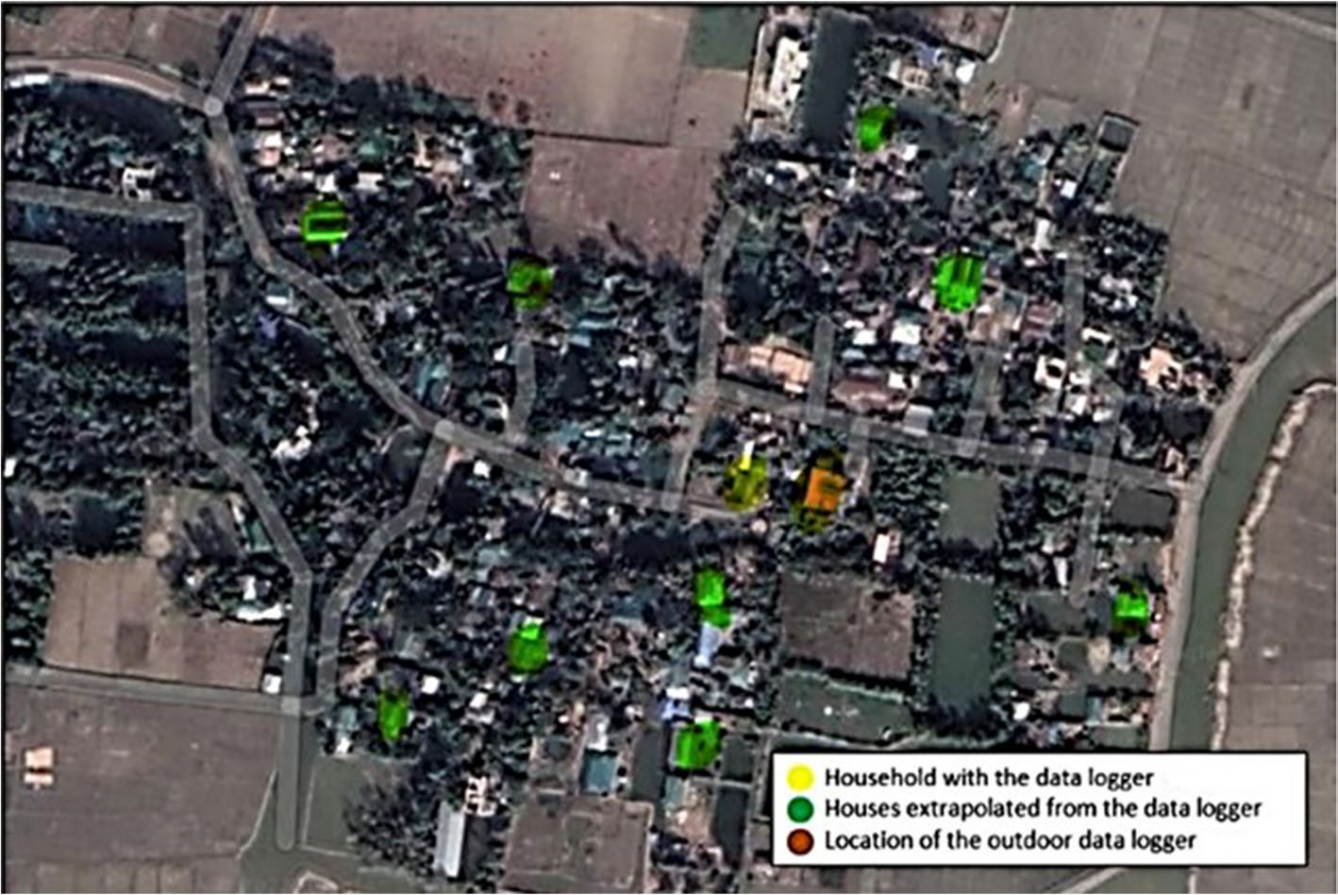
Example map of a village in Chakaria. The locations of indoor and outdoor data loggers are shown. One indoor data logger, in yellow, is extrapolated into 9 other houses (in green) with the same materials. There is one data logger placed outdoors

HL-1D data loggers by rotronic© were used to measure temperature and humidity every hour during the study period of 16th of March to the 8th of May, 2022. Each village had one data logger placed in a house in the centre of the village of the same material as the other 9 chosen for that village (Fig. 2). Six outdoor data loggers were in the study area, 2 close to the coast, 2 in the center and 2 in the more hilly inland area. The outdoor data loggers were placed in public spaces like mosques or schools under a roof, and without direct contact with any surface or in the direct sunlight. Indoors loggers were placed 1- 2 m above the floor level, not in direct contact with any wall or exposed to sunlight. All workers were given specific training and a detailed protocol including a problem-solving section. They were installed between the 9th and 15th of March, 2022.

#### Environmental parameters

The hourly temperature and relative humidity information collected by the data loggers were used to calculate the hourly Heat Index. The Heat Index (HI [°C]) is a well-known measure of the apparent temperature felt by the body which is often used to describe events of heat stress [11]. The data collected by the data logger inside the central house was applied to all the other houses in that village which had been previously selected to have the same housing materials. The average distance between the logger-installed houses and the other selected houses for extrapolation (9 houses for each logger) in the 49 villages is approximately 90 m. Environmental parameters were standardized starting on the first date that all loggers were recording and ending on the first date that a data logger was collected which was from the 16th of March to the 8th of May, 2022. This time of year, was chosen as it’s the hottest months of the year. To understand the effect of household characteristics on temperature, the model was made on the 49 houses which had the data loggers, it was then verified with the whole data set.

#### Questionnaire

A questionnaire was designed with the aim of capturing the feelings of illness of the household members in the last month as well as collecting information on the household characteristics. The questionnaire was designed with a stepwise approach of round table discussions. Input from a variety of backgrounds including urban sciences, health policy and qualitative research was taken to address the different high-risk behaviours. The final questionnaire consisted of four sections and 82 questions. It took approximately 30 min to complete. Consent was taken prior to the delivery of the questionnaire by all participants. This consent form was signed by the respondent as well as a witness for identity confirmation. Some questions were identical to those in the HDSS for verification.

To ensure reliable data collection, the health-skilled workers were formally trained for 3 days prior to the start of the survey. For the pre-field test, each health worker carried out 3 questionnaires which were then reviewed for inconsistencies and discussed in a group setting to clarify any doubts. The questionnaire was delivered with an online platform, ODK, on digital tablets. The same existing team of 10 women conducting the HDSS did the questionnaire.

### Data processing and variable definition

Before the analysis, data was reviewed and cleaned. As the households were part of the HDSS, the information could be verified for correctness, in those cases where mismatches were found, the health worker was sent to verify.

The wealth index, constructed from household asset data using principal component analysis was used to categorise households into socioeconomic groups. Households were categorised into five socioeconomic quintiles: lowest, second, middle, fourth, and highest. All odds ratios and relative risk metrics have been adjusted for the wealth index.

The outcome variables were the reports of symptoms. Exposures were the indoor household maximum temperature and household characteristics. Socio-economic status was considered a confounder.

To investigate the factors that affected indoor characteristics: average and maximum temperatures, humidity, and heat indices; the household characteristics were modelled.

Regarding the factors which affected respondent heat coping behaviours, their personal characteristics as well as the household conditions and indoor environment were considered. When analysing the factors affecting health outcomes, the dependent variables were occurrence of disease episode (Yes/No) and number of episodes of each type per household. It was analysed at person level as well to investigate the effect of their personal behaviours. For that section the independent variables were indoor environment, household conditions and heat coping behaviours.

Symptoms were grouped in 4 categories and presented in Table 1. Participants were asked to report the symptoms that everyone in their household had had in the past month.

**Table 1:** Classification of acute symptoms of health problems

	Category of health problems	Symptoms
1	General	Fever, body pain, headache, and malaise/fatigue
2	Respiratory	Runny nose, sore throat, cough and breathing difficulties
3	Digestive	Diarrhoea, nausea, vomiting, and stomach pain
4	Non-specific	Skin dryness, local site infection, skin itchiness and eye conditions

**Fig. 3:**
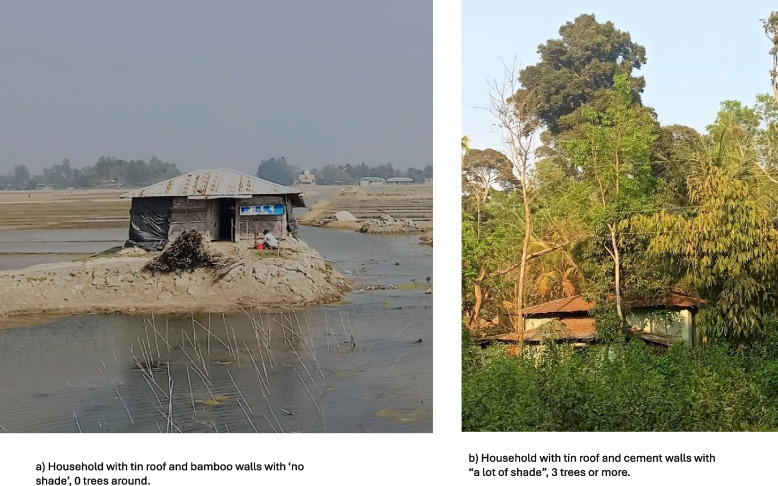
Images of the surrounding area around two households from the study to show shade and household material

The material of the house and the amount of shade was directly observed by the health worker who counted how many trees or buildings gave shade. Shade was defined in 3 categories, 0 was “no shade”, 1–2 was “some shade" and 3 or more were “a lot of shade”. Context for household characteristics can be found in Fig. 3. The household members’ ages in years were stratified as 0–5, 6–18, 19–30, 31–59, over 60. The average of the daily maximum indoor temperature for the study period was calculated for each household containing a data logger. The households with higher than the average daily maximum temperature during this time were classified as “hotter houses” and households with lower than that threshold were labelled as “colder houses”.

### Statistical analyses

Statistical analyses were conducted using R version 4.1.2. Graphs and figures were made using Office package and R. Bivariate associations were assessed using Pearson chi-squared test, variables were transformed into bivariate as previously mentioned to allow for the analysis with multilogistic regression. All statistical tests were two tailed and were considered statistically significant at less than 0.05. Chi-squared test of association was applied to determine whether disease counts differed between hotter and colder houses.

Two secondary households (without a data logger) had household materials that did not correspond to either bamboo or cement and they were removed for the temperature modelling but included for the coping behaviours analysis.

## Results

### Sociodemographic characteristics of the population

Most (416, 84.9%) of the respondents to the survey were not the household head. Males made up 431 (88.0%) of household heads with an average of 47.8 ± 13.4 years old (20 to 86). Respondents' average age was 37.4 ± 11.1 (17 to 81) years old, with an average of 5.6 ± 3.74 years of education. One-fifth (104, 21.2%) had no schooling and 79 (16.1%) had completed higher education.

In 405 (82.7%) of the households, women did not have remunerated work. The average household income was 19,844 ± 13,846.1 Tk per month (211.4€ ± 147.5€ in April 2022, time of the study) with a range from 3,000 to 90,000 Tk.

### Environmental factors

The daily minimum temperatures ranged from 17.5 °C to 31.4 °C and the daily maximum temperatures from 27.7°C to 42.1°C. The values for minimum and maximum values for relative humidity during the study period were 23.6% and 99.9% respectively.

The average indoor HI values obtained were from 25.5°C to 44.4°C, the minimum values for the study period ranged from 17.6°C to 39.5°C and the maximum values from 29.0°C to 54.8°C.

Overall, maximum temperatures were about 2 degrees lower indoors than outdoors (*p* < 0.001), but average temperatures were higher indoors (*p* < 0.001). HI followed a similar pattern, maximum HI outdoors were higher than indoors, 42.0°C compared to 40.1°C (*p* < 0.001) and average HI were higher indoors than outdoors.

**Fig. 4:**
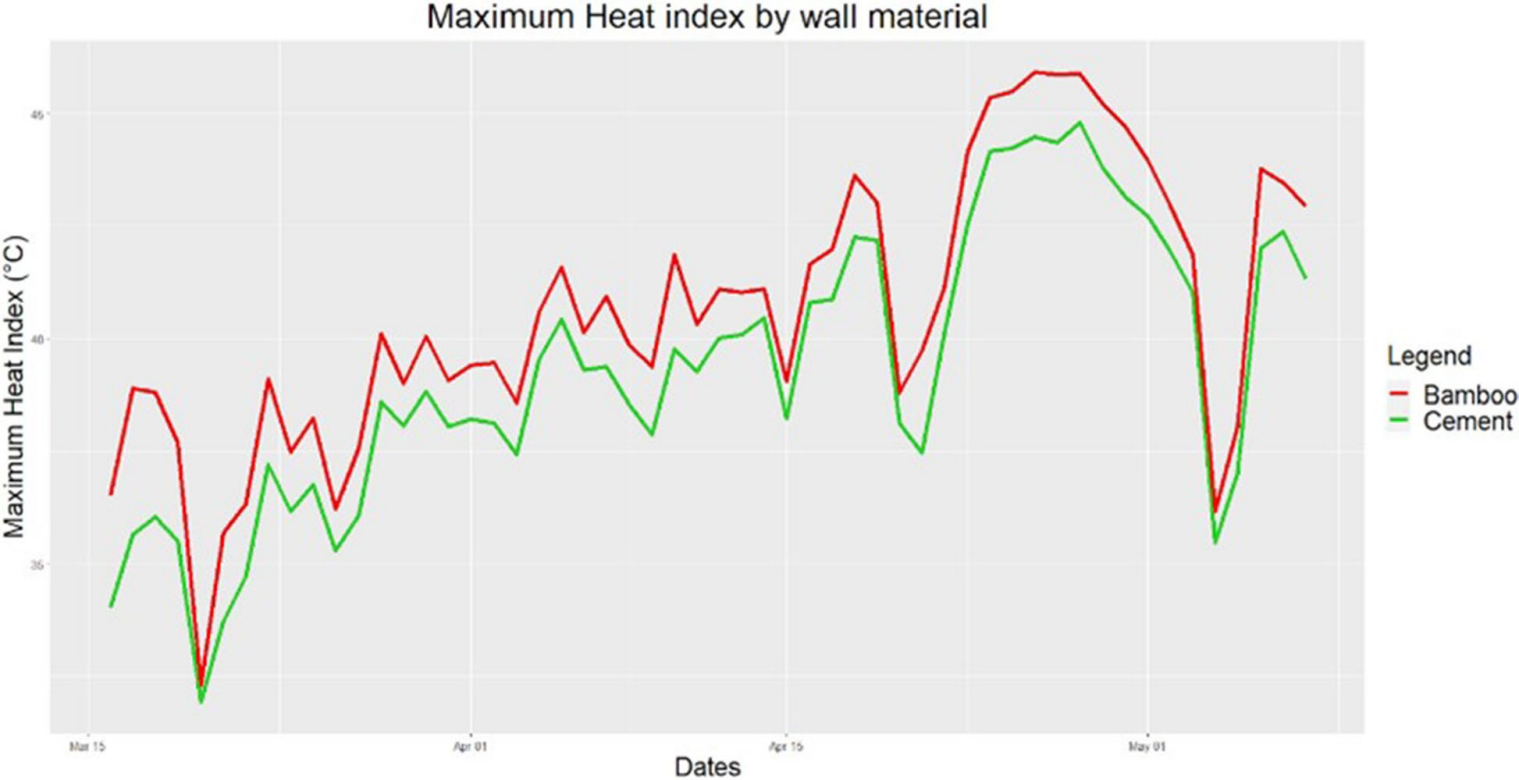
Variation in maximum HI indoors by wall material. Houses made from bamboo showed consistently higher HI than those from cement

**Fig. 5:**
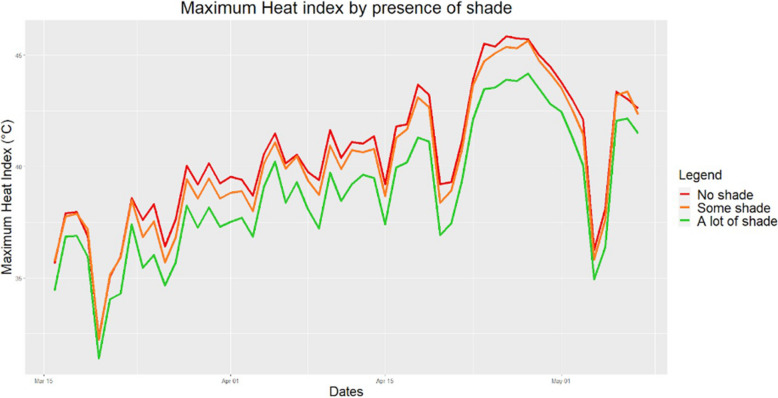
Variation in maximum HI indoors by the presence of shade. Houses with no shade showed consistently higher HI than those with some or a lot of shade. Shade was defined in 3 categories, 0 was “no shade”, 1–2 was “some shade" and 3 or more were “a lot of shade”

Approximately two-thirds of houses had bamboo walls and one-third had a cement or brick wall. Having a false ceiling was common, as 73.5% of houses had it. 67.8% of households had shade from at least one plant. Regarding protective factors, having a cement wall decreased the HI compared to having walls made of bamboo (Fig. 4). Moreover, the presence of shade increased humidity, but it significantly decreased HI indoors (Fig. 5). Having 3 or more trees had a significantly greater effect than having less than 3. False ceiling, did not have a conclusive effect on HI. All 490 houses in Chakaria had tin roof; this was purposely selected.

### Symptoms reported

There were a total of 2519 people living in the 490 households surveyed. There was at least one person sick in 315 (64.3%) of houses. During the months of April and May, 570 (22.6%) people reported feeling sick, with a total of 1,084 symptoms. Vulnerable populations were children and elderly, which were defined as under 5 years old and over 60, and made up 173 (30.4%) and 76 (13.3%) of people sick respectively.

Fever was amongst all categories the most reported symptom. When stratified by age, general symptoms had the lowest rate of reporting in children under 5, with the average age being 36.7 ± 11.7 years old (Table 2).

**Table 2:** Counts of reported symptoms, blue is respiratory, orange is digestive, green is non-specific and general is in purple. The duration of each symptom is given in days and the age of the patient in years, in a mean +—SD format. People living in bamboo and cement houses include all people living there irrespective of whether they reported illness. Similarly for men and women, it includes all people living in the houses. There are 2 households who account for 5 people ill whose houses did not fall under bamboo or cement wall

	**Number of cases reported**	**% With treat**	**Dur. (Days)**	**Age (Years)**	**% Women**	**% Men**	**% People in Bamboo houses**	**% People in Cement houses**
Total (n)	1062				1266	1253	1575	933
General								
Fever	348	91%	5.9 ± 4.5	18.2 ± 18.9	210(16.6%)*	138 (11.0%)*	244 (15.5%)*	103 (11.0%)*
Headache	55	80%	5.8 ± 5.4	38.2 ± 17	45 (3.6%)*	10 (0.8%)*	30 (1.9%)	24 (2.6%)
Body ache	48	88%	11.5 ± 7.9	39.0 ± 20.2	26 (2.1%)*	22 (1.8%)*	31 (2.0%)	17 (1.8%)
Fatigue	29	93%	5.8 ± 5.3	39.2 ± 17.8	26 (2.1%)*	3 (0.2%)*	23 (1.5%)*	5 (0.5%)*
Respiratory								
Runny nose	169	89%	5.8 ± 4.4	17 ± 17.7	109(8.6%)*	60 (4.8%)*	116(7.4%)*	51(5.5%)*
Dry cough	153	87%	6.6 ± 5.3	19.1 ± 19.9	88 (7.0%)	65 (5.2%)	101 (6.4%)	50 (5.4%)
Sore throat	21	91%	6.6 ± 4.2	23 ± 16.2	16 (1.3%)*	5 (0.4%)*	13 (0.8%)	8 (0.9%)
B. difficulty	16	94%	9.1 ± 5.8	25.6 ± 22.8	8 (0.6%)	8 (0.6%)	10 (0.6%)	5 (0.5%)
Digestive								
Diarrhoea	68	94%	4.5 ± 5.8	23.9 ± 19.7	26 (2.1%)*	42 (3.4%)*	38 (2.4%)	30 (3.2%)
Vomiting	29	100%	2.4 ± 2	15.5 ± 15.1	11 (0.9%)	18 (1.4%)	21 (1.3%)	8 (0.9%)
Stomach-ache	18	100%	4.8 ± 6	30.5 ± 17.9	3 (0.2%)	15 (1.2%)	15 (1.0%)	5 (0.5%)
Nausea	8	100%	3.8 ± 3.9	29.5 ± 16.2	4 (0.3%)	4 (0.3%)	6 (0.4%)	1 (0.1%)
Non-specific								
Skin itch	49	94%	11.7 ± 7.5	12.2 ± 17.2	27 (2.1%)	22 (1.8%)	33 (2.1%)	15 (1.6%)
LSI	40	88%	14.1 ± 9.5	16.2 ± 19.8	25 (2.0%)	15 (1.2%)	30 (1.9%)	9 (1.0%)
Eye condition	11	100%	15.9 ± 10.4	36.3 ± 30.3	7 (0.6%)	4 (0.3%)	9 (0.6%)	1 (0.1%)

*Dur* Duration, *LSI* Local site infection, *W/ treat* With treatment

(*) difference in the category is significant at 0.05

Runny nose and dry cough made up 319 (89.7%) of respiratory symptoms 169 (47.1%) and 153 (42.6%) respectively. However, when occurring, breathing difficulties lasted the longest on average and more often needed treatment (Table 2).

Digestive symptoms were less common than the respiratory ones. Diarrhoea was the most prevalent one 68 (55.3%). Almost half of vomiting cases were suffered by children under 5. Treatment was required in all except 4 cases of digestive disease (Table 2).

As for the non-specific categories of symptoms, it was mostly children who suffered from skin conditions, either skin itch like measles, or local site infections. Non-specific symptoms lasted for longer than other categories of symptoms (Table 2).

Overall, women were found to have significant higher odds of reporting symptoms than men (AOR = 1.4, *p* < 0.01). When looking specifically at the symptoms the odds were higher for most symptoms. For body ache, the odds were notably higher (AOR = 1.02, *p* < 0.0001), and similarly for headache (AOR = 1.40, *p* < 0.0001), fatigue (AOR = 2.00, *p* < 0.001), runny nose (AOR = 1.65, *p* < 0.01), and sore throat (AOR = 2.74, *p* < 0.05). On the other hand, the odds of reporting fever were moderately higher in women (AOR = 1.42, *p* < 0.01). However, diarrhea was less common in women compared to men (AOR = 0.53, *p* < 0.05).

Most respondents (352, 71.9%) considered Summer (Apr.–May) a period where their family got sick most often. The second most common season was Winter (Dec.–Jan.), considered by 204 (41.7%).

### More disease reports in hotter houses

**Fig. 6:**
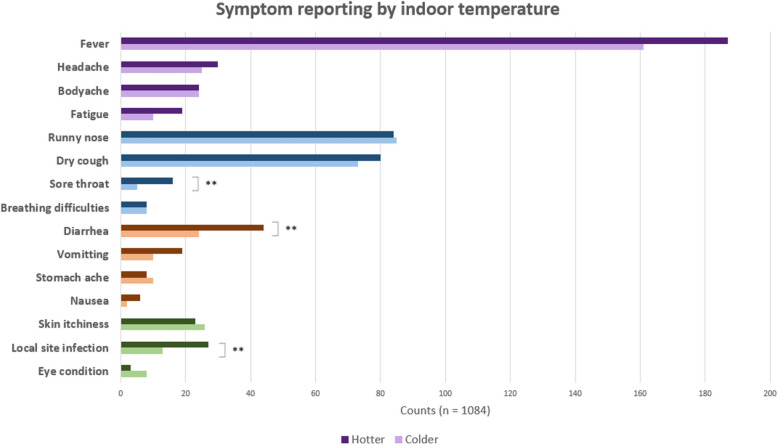
Symptom reporting by indoor temperature, symptoms are colour coded by category. Sore throat, diarrhea and LSI occur significantly more often in houses with higher maximum temperatures. Hotter houses are those with higher than average daily maximum temperatures and colder and those with lower daily maximum temperatures than average

To assess the direct effect of temperature on feelings of illness, houses were divided into either hotter or colder than the average maximum temperatures. Houses with hotter maximum temperatures had more counts of all symptoms except for eye conditions, skin itch, runny nose and stomach-ache which had more counts in colder houses. However, only sore throat and diarrhoea occurred significantly more often in hotter houses which might be because of a lack of power. Hotter houses had 3.2 times higher likelihood of getting a sore throat (CI:1.1–11.6, *p* < 0.05) and 1.9 times more likely to have diarrhoea (CI:1.1–3.5, *p* < 0.05) (Fig. 6).

Houses with maximum temperatures which were hotter than average took more treatment than colder houses 92.2% compared to 88.1% (*p* < 0.05). Age did not play a role on whether they were more affected by the heat.

### Relationship between household characteristics and health

The material of the households was protective to health. In households with cement walls, the odds of reporting any symptom were significantly lower by 31% compared to those with bamboo walls (AOR = 0.69, *p* < 0.01). There were differences regarding specific symptoms as well. Namely, fever, fatigue and runny nose were all significantly less reported in cement houses compared to those with bamboo walls, with the odds being 42% lower (AOR = 0.58, *p* < 0.001) for fever, 70% (AOR = 0.3, *p* < 0.05) for fatigue and by 44% (AOR = 0.56, *p* < 0.05) for runny nose (Table 2). Additionally, in households with cement walls, the expected number of symptoms each person reported when ill was significantly lower by 18% compared to those with bamboo walls (RR = 0.81, *p* < 0.05), meaning less complex diseases in cement houses.

Another protective factor was having shade. In areas with no shade, the expected symptom count was significantly higher by 27% compared to areas with a lot of shade (RR = 1.27 *p* < 0.01, while areas with some shade showed no significant difference compared to a lot of shade.

### Heat coping behaviours

The study also aimed to assess if women, who were considered at high risk for spending long periods of time indoors, living in this area performed heat coping behaviours. Most women performed some heat coping mechanisms. Over 90% of respondents reported that they showered, turned the fan on and drank more in response to heat. There were 414 (84.5%) women who stated that they went outside their home. It was less common to change clothing with 270 (55.1%) and 112 (22.9%) reporting they wore lighter clothes or took clothes off respectively (Table 3).

**Table 3:** Compliance with heat coping behaviours in vulnerable women in Chakaria. Odds ratio and 95% CI of having reduced counts of disease if performing this behaviour

	**% Women performing behaviour**	**Odds Ratio**	**95% CI**
Showering	92.5%	1.1	[0.5, 2.6]
Going outside	84.5%	1.5	[0.8, 2.9]
Turning fan on	93.7%	0.7	[0.3, 1.7]
Wearing lighter clothes	55.1%	0.8	[0.5, 1.1]
Removing clothing	22.9%	0.9	[0.6, 1.5]
Drinking more	90.2%	1.3	[0.6, 2.7]

Of the 477 households in Chakaria with female respondents, 141 of them were sick during the study period. Those houses were subdivided to check whether the behaviours reported played a role on whether they got sick the last month or not. Respondent heat coping behaviours did not play a significant role on whether they got sick during the previous month or not (Table 3). Possible reasons for this are provided in the discussion.

## Discussion

In Bangladesh, vulnerable populations are already suffering from extremely high temperatures which are likely to worsen with continued global warming. This study showed that, in resource-poor settings of Chakaria, outdoor and indoor temperatures are comparable and at times even higher indoors. The present study showed that they often exceeded the WHO threshold of comfort for people in hot climates of 29.8°C [12]. Nevertheless, living in a hotter house is not only a matter of decreased comfort. Households with higher maximum indoor temperatures in this study reported more cases of almost all recorded symptoms than cooler houses, suggesting that there might be an association between high indoor temperatures and disease.

Specifically, people reporting cases of diarrhea were significantly more likely to live in hotter houses. Gastrointestinal infections caused by bacteria have been shown to increase with higher temperatures [13]. Some bacteria causing food-borne diseases like Salmonella have more rapid replication rates at higher temperatures, and outbreaks at city level are associated with weekly maximum temperatures [14, 15]. This study highlights that the microclimate at home can also play a role. Additionally, cases of sore throat were also more common in hotter houses. Respiratory viruses are commonly associated with colder temperatures and low humidity, however, high daily fluctuations, which were also found in this study’s hotter houses, are associated with higher cases of bacterial respiratory infections [16]. This could explain why this study and others have found an increase in respiratory infections with higher temperatures [3].

More people in the hotter houses reported taking treatment for their illness than in the less hot houses. The need for treatment can be considered a proxy for the severity of disease, therefore, this suggests that more symptoms reported in hotter houses were serious. It has been reported that 90% of treatment in rural Bangladesh is taken from unqualified providers and according to a recent study about 80% of the drugs prescribed by them are inappropriate or harmful [7]. Ensuring health care is accessible for all is essential for appropriate disease management of vulnerable populations in Bangladesh.

To prevent the negative effect of high indoor temperatures on human health, one can reduce people’s vulnerability or increase adaptive capacity [17]. Reducing vulnerability uses mitigation policies which aim to make the challenge less severe. For example, limiting the increase of indoor temperatures through the modification of housing characteristics [18]. To increase adaptive capacity, coping behaviours can be used to facilitate adjusting to the existing problem (high temperatures). The following subsections will explore these two possibilities.

### Housing materials to reduce vulnerability

Poverty makes people vulnerable to climate because they cannot afford to increase their mitigative capacity [19]. Housing is the first layer of “defence” from the outside world, and thus, it must be adapted to protect from the local challenges. The houses in this study had a tin roof with either bamboo or cement wall. Bamboo houses are not well adapted, as due to sea-level rise, Chakaria is a flood prone area, and this study showed that houses made of bamboo were hotter and reported fever and fatigue more often than cement houses.

Nevertheless, changing bamboo houses into cement is hard to envision in the short term because it goes hand-in-hand with a higher economic status and stability for long-term investments. However, this study highlighted that simpler options like having shade around the house decreased indoor temperatures. Having trees around a house can decrease the surface temperature by up to 11- 15°C at peak temperatures and improve air quality by sequestering CO2 [20, 21]. Moreover, having shade reduced the number of symptoms reported, which could be due to the reduction of indoor temperature or an overall healthier environment. In this study, all houses had tin roofs, other simple strategies to reduce heat absorption have been studied, one successful option was painting the tin roofs white. This initiative has started in Dhaka and the first results show a reduction of 3.5°C indoors after coating [22, 23]. Other options exist like Jutin, which has 5000 times less heat conduction than aluminium and is resistant to salt water (unpublished report of ICDDR,B). It is currently being implemented in vulnerable areas like the Rohingya migrant camps, an area close to Chakaria [24, 25]. It is essential that future research explores some of the above-mentioned interventions to find real solutions to increase climate resilience that focus on changes to housing which are affordable, easy to implement, and culturally acceptable.

### Heat coping mechanisms to increase adaptability

Coping behaviours are efficient adaptation strategies to extreme heat, they include drinking water, changing food storage habits, using a fan, showering or removing clothing. This study focused on whether women performed them as they spent longer exposed to indoor temperatures. However, no association was found between women performing or not certain coping behaviours and experiencing illness. Many of the respondents reported similar coping behaviours and a bigger sample size could be needed to see an effect. Houses are not independent; views, traditions and at times even facilities are shared amongst them leading to similar behaviours. Additionally, it is important to verify compliance to the behaviours reported as participant bias could have meant women reported the “correct” behaviour despite not performing it. Not performing one behaviour could have been compensated with performing another one since all women reported doing at least two of the six behaviours. Despite there being no direct effect on disease outcomes, these behaviours are likely to decrease feelings of heat. These mitigating behaviours have to be emphasized, because people living in the tropics are predicted to reach the physiological limit to heat adaptability more and more often [26].

This study addresses an understudied area by exploring the role of housing in climate preparedness to ensure healthy lives. To better inform policy, future research should investigate whether certain coping behaviours have an especially protective effect against illness. Additionally, a larger sample size is needed to determine if certain diseases or symptoms are more strongly associated with high indoor temperatures. Finally, assessing the external validity of these findings across different high-temperature settings would help determine their broader applicability.

### Limitations and strengths

The results must be interpreted in light of several limitations. Firstly, the reports of symptoms were self-reported which is prone to many biases. Recall and social desirability bias are especially notable, symptoms were reported for the last month, recall bias could be reduced by shortening this timeframe. Prompting bias can also be considered as fever, the first symptom in the questionnaire, was the most reported one. Nevertheless, self-reported information is very valuable and provides subjective information about a patient’s feeling of well-being. Additionally, respondents also reported health conditions of other household members and thus, some extent of under-reporting cannot be ignored. This could explain why overall in this study women, who made up most respondents, suffered from more symptoms. Lastly, as stated previously, every region has different coping mechanisms and housing characteristics, and caution is advised when generalizing the results.

Despite these limitations, the study has several relevant strengths. The study location is an iccdr’b HDSS area which allows facilitated access and follow-up of the population. Previously, most research on indoor temperatures focused on developed areas and housing with good insulation, however, this study focused on vulnerable populations with little heat mitigation options. Moreover, research is lacking on the specific challenges of women, so focusing on them allows more targeted and specific measures to be put in place. Lastly, indoor temperature and humidity values were taken on an hourly basis and high accuracy could be obtained on daily variations.

## Conclusion

The present cross-sectional study explored the relationship between indoor temperatures and acute symptoms in vulnerable populations of Bangladesh. It found that suboptimal housing conditions with inappropriate materials which increase heat can have an impact on health. People living in hotter houses reported more cases of diarrhoea and sore throat. Shade and having cement houses significantly decreased indoor temperature in this study and should be considered as options to mitigate increasing temperatures. This was namely the case with fever and runny nose which were less commonly reported in cement houses. Studies with larger sample size and a longer follow up period should be carried out to gain further understanding into these mechanisms. Moreover, this study showed that despite not protecting against illness, Bangladeshi women’s heat coping behaviours were overall good. However, compliance should be verified by studying the behaviours directly throughout the year and with a larger sample size. Nevertheless, the health of vulnerable populations is a multi-layered challenge which cannot be solved with only one measure. As such, close attention should be paid to prioritizing strategies which can be easily implemented in low-resource settings.

## Data Availability

The raw data supporting the conclusions of this article will be made available according to icddr,b’s data access policy.
